# Stakeholder-informed priorities for process improvement in Warfighter Brain Health efforts

**DOI:** 10.3389/fpubh.2026.1815983

**Published:** 2026-05-21

**Authors:** Rebecca A. Ivory, Ricky Ditzel, James S. Meabon

**Affiliations:** 1 School of Nursing, Emory University, Atlanta, GA, United States; 2 School of Nursing, University of Delaware, Newark, DE, United States; 3 Rush University School of Medicine, Chicago, IL, United States; 4 VA Northwest Mental Illness Research Education and Clinical Center (MIRECC), VA Puget Sound Health Care System, Seattle, WA, United States; 5 Department of Psychiatry, School of Medicine, University of Washington, Seattle, WA, United States

**Keywords:** blast overpressure, community engagement, decision support, exposure tracking, low-level blast, mild traumatic brain injury, quality improvement, special operations forces

## Abstract

**Introduction:**

Blast overpressure exposure and mild traumatic brain injury (mTBI) remain important concerns within Warfighter Brain Health efforts, yet practical gaps persist in symptom recognition, education, and care navigation.

**Methods:**

An anonymous web-based survey of U.S. Special Operations Forces (SOF)-affiliated stakeholders was conducted from approximately 15 April to 15 June 2025 (N = 64). Items addressed community issues, symptom priorities, publication audiences, tool priorities, and preferred online formats. Analyses were descriptive; ranking items were additionally summarized using a secondary weighted priority score.

**Results:**

Respondents most often endorsed medical knowledge deficits, limited access to specialized care, and difficulty obtaining timely blast- or mild-TBI-related information. Weighted descriptive rank aggregation supported high priority for attention/concentration and decision-making changes, depressed mood and isolation, and suicidal thoughts and behaviors. Clinicians and medics were the leading dissemination audiences, and self-administered neurocognitive baseline, symptom monitoring, and event/exposure tracking tools were the leading tool priorities. Brief videos and personal testimonials were the most preferred online formats.

**Discussion:**

In this convenience sample, findings support actionable priorities focused on strengthening clinical competency, improving information accessibility, and developing tools for assessment, tracking, and symptom management, supporting Department of Defense brain health objectives.

## Introduction

Blast overpressure (BOP) exposure from weapons system use has become a central concern within Department of Defense / War (DoD/W) Warfighter Brain Health efforts intended to preserve performance, readiness, and long-term health outcomes (1). The DoD/W Warfighter Brain Health Initiative emphasizes proactive approaches to brain health, including attention to blast overpressure alongside concussion and other brain injuries (1). Although the literature on repetitive blast exposure continues to evolve (2), military cohort studies have reported symptom burden (3), biomarker changes (4–6), and neurocognitive changes (7) that support better surveillance, education, and standardized care pathways (8–11).

At the same time, implementation of practical and trusted resources remains constrained by variability in provider familiarity with blast-related concerns (10), uneven access to specialized services (12), and inconsistent data integration across the military-to-veteran transition (9). Within this context, stakeholder feedback can help identify which problems, audiences, and tools should be prioritized first for process improvement efforts.

The present project was designed as a stakeholder-engaged, community-informed needs assessment to support near-term quality improvement in Warfighter Brain Health-related education, symptom monitoring, and care navigation. Rather than estimating prevalence or testing causal hypotheses, the goal was to identify and describe which issues and practical needs were most frequently prioritized by respondents connected to U.S. Special Operations Forces (SOF) communities (e.g., U.S. Navy SEALS, U.S. Army Special Forces). We therefore conducted a brief anonymous survey of SOF-affiliated stakeholders to describe their priorities across symptom domains, target audiences, and tools that could inform future resource development and implementation planning.

## Methods

### Study framework and design

This project was undertaken as a stakeholder-engaged community feedback and quality improvement (QI) activity intended to inform practical improvements in Warfighter Brain Health-related processes, rather than to test epidemiologic hypotheses. The report is presented in a conventional scientific format for readability, but the work is most appropriately interpreted as a descriptive needs-assessment aligned with community-engagement and QI principles. During approximately 15 April to 15 June 2025, the study team administered a brief, anonymous, web-based survey (see Supplementary material) through providing established networks connected to SOF-affiliated individuals and stakeholders (13, 14) a shareable link to the survey. Survey recipients were encouraged to further share the link with their known adult, SOF community stakeholders which included but were not limited to Servicemembers, Veterans, affiliated Caregivers, and family. No reminders to complete the survey were sent. Because recruitment was convenience-based and network-mediated rather than probability-based, the sample was not designed to generate representative estimates for the broader SOF population.

### Participants and survey completion

The analytic sample comprised 64 completed survey responses. No directly identifying information was collected. The survey captured military status, role, sex, service branch, and self-described occupational specialty or rating where respondents elected to provide it. Age was not collected, and the survey did not objectively verify blast-exposure history, mTBI history, or clinical diagnoses—none of which can occur through survey-based mechanisms. Accordingly, the present findings should be interpreted as stakeholder perspectives on blast- and mTBI-related priorities among respondents connected to military and SOF settings, rather than as exposure-verified or diagnosis-verified subgroup estimates.

Because age was not captured, this study cannot characterize the age distribution of respondents. Survey items were not mandatory, the analysis was not restricted to completed responses and partial responses were possible and accepted for analysis.

### Survey development and measures

The survey was purpose-built over an approximately 2-month period to support a pragmatic stakeholder-engagement and quality-improvement needs assessment focused on blast exposure, mild traumatic brain injury, and Warfighter Brain Health priorities in SOF-affiliated communities. Instrument development was iterative and informed by informal semi-structured background interviews, ongoing stakeholder consultation, and repeated draft review. The core review group included approximately 22 stakeholders with relevant lived or professional expertise, including current or former U.S. Special Operations Forces Servicemembers, several with prior Special Operations Combat Medic (SOCM) experience or medical training, and an interdisciplinary panel of expert clinician-scientists and allied health professionals spanning neuroscience, psychiatry/psychology, physical therapy, occupational therapy, internal medicine, and endocrinology. Initial interviews with SOF Servicemembers and additional affiliated respondents were used to identify commonly voiced community concerns, including perceived acute and long-term effects of blast exposure, barriers to care, information needs, and priorities for education and symptom-management tools. Draft survey items and response options were then refined through repeated email- and phone-based review focused on content relevance, clarity, terminology, reading level, formatting, and organization. Additional feedback was obtained from SOF caregivers and other non-Servicemember community members to improve accessibility and ensure that the instrument reflected concerns salient beyond current Servicemember respondents alone. The instrument included respondent characteristics; a ‘select four’ item regarding major community issues related to blast overpressure and mild traumatic brain injury; a multi-select item on preferred formats for online educational content; and three ranking tasks addressing symptom-domain priorities, audiences for publication or dissemination, and practical tools to support decision-making and monitoring. The final survey was designed to maximize face validity, content relevance, and practical actionability for stakeholder-informed prioritization rather than psychometric measurement.

### Analysis

Given the modest convenience sample and the practical QI purpose of the project, analyses were intentionally descriptive. Categorical variables were summarized as counts and percentages. For ‘select all that apply’ items and the ‘select four’ item, we report the percentage of respondents endorsing each option (percentages do not sum to 100%). For ranking items, we first examined the distribution of ranks by option and qualitatively identified options most frequently placed near the top ranks.

Ranking items were also summarized using a normalized Borda-style weighted priority score (higher scores indicate higher priority), mean rank, and rank-1 count. Because the respondent-level surveys are not available, counts for the displayed rank distributions were reconstructed from the aggregate data using axis-calibrated bar-height estimates constrained so that each item summed to the number of question respondents across ranks. These secondary analyses are intended to strengthen descriptive prioritization, not to support inferential comparisons.

## Results

### Respondent characteristics

Most respondents were active-duty service members (31/64, 48.4%) or Veterans with SOF backgrounds (23/64, 35.9%). The majority identified as male (56/64, 87.5%), with representation across the U.S. Navy (36/64, 56.3%), Army (22/64, 34.4%), Air Force (5/64, 7.8%), and Marine Corps (1/64, 1.5%). Overall, 79.7% identified as current or former SOF, while additional respondents identified as SOF enablers (10.9%), spouses or partners (10.9%), caregivers (6.3%), and/or other stakeholders (10.9%). Self-entered military occupational specialties and ratings were heterogeneous and included combat medics, operations sergeants, Special Warfare Combatant-craft Crewmen, and Navy SEALs.

Because recruitment relied on established networks and because some stakeholder categories were represented by relatively few respondents, the sample should be understood as a convenience sample of SOF-affiliated stakeholders rather than a representative cross-section of all SOF personnel, Veterans, families, or caregivers.

### Priority issues, audiences, and tools

**Figure 1:**
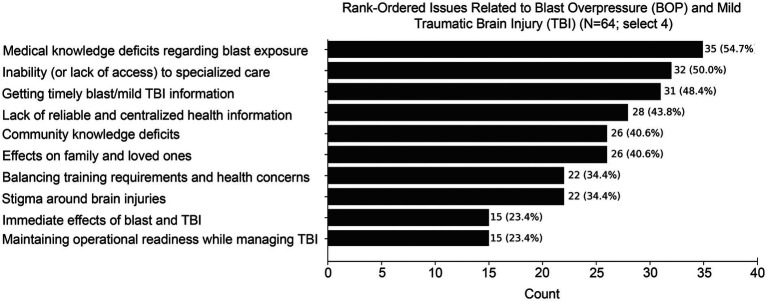
Stakeholder-endorsed issues related to blast overpressure and mild traumatic brain injury (TBI) among U.S. Special Operations Forces (SOF)-affiliated respondents. Bar graph represent counts (with percentage of respondents) for endorsements of the top four issues within stakeholder communities related to blast exposure and mild TBI. *N* = 64; select 4 issues.

The most frequently endorsed issues related to blast overpressure and mild traumatic brain injury were medical knowledge deficits regarding blast exposure (35/64, 54.7%), inability or lack of access to specialized care (32/64, 50.0%), and difficulty obtaining timely blast- and mTBI-related information (31/64, 48.4%) (Figure 1). Respondents also commonly endorsed lack of reliable and centralized health information (28/64, 43.8%), community knowledge deficits (26/64, 40.6%), and effects on family and loved ones (26/64, 40.6%). Stigma around brain injuries and balancing training requirements with health concerns were each endorsed by 22/64 respondents (34.4%).

**Figure 2:**
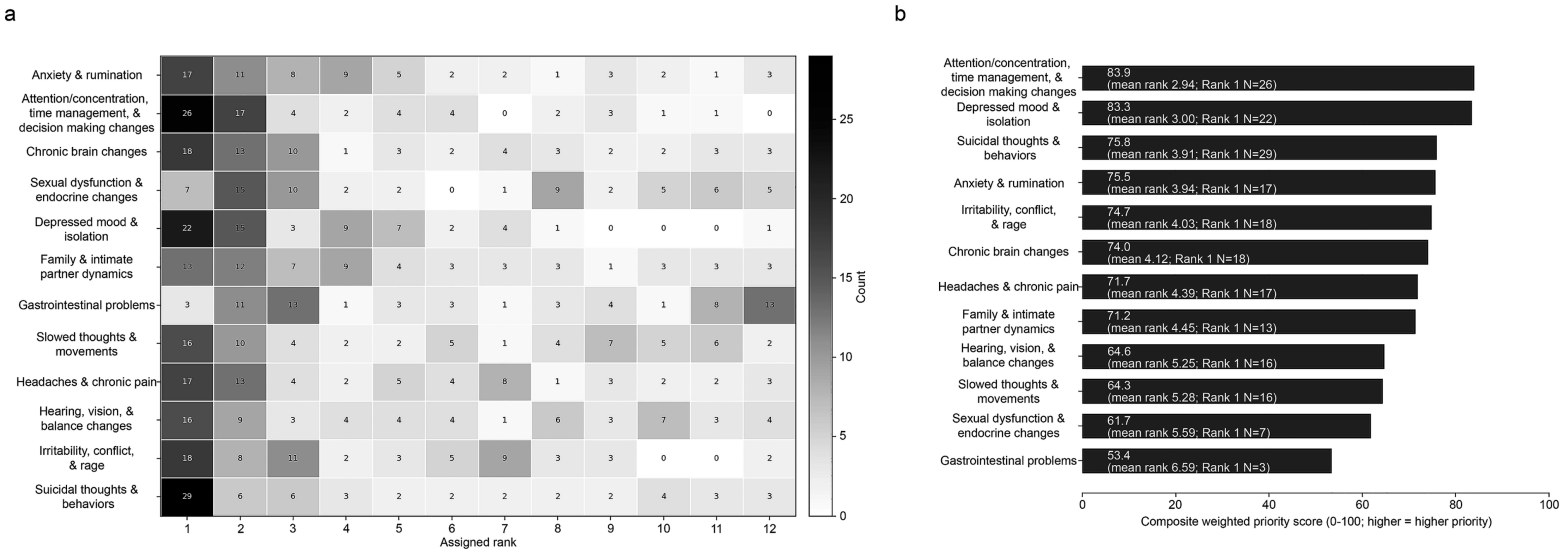
Stakeholder-endorsed symptom priorities related to blast overpressure and mild traumatic brain injury (mTBI) among U.S. Special Operations Forces (SOF)-affiliated respondents. **(a)** Symptom rank distribution heatmap (Rank 1 = highest priority). Grayscale annotated heatmap shows rank counts across 12 symptom domains. **(b)** Weighted symptom-priority summary for the 12 blast/mTBI symptom domains, displayed as a normalized Borda-style composite priority score, with higher values indicating higher priority; labels report mean rank and rank-1 counts. *N* = 64; respondents selected up to four items.

In the symptom-domain rankings, the items most consistently appearing near the top included suicidal thoughts and behaviors; attention, concentration, time management, and decision-making changes; depressed mood and isolation; irritability, conflict, and rage; and chronic brain changes (Figure 2a). Secondary weighted descriptive aggregation of the symptom ranks yielded the highest composite priority scores for attention/concentration and decision-making changes (83.9; mean rank 2.94), depressed mood and isolation (83.3; mean rank 3.00), suicidal thoughts and behaviors (75.8; mean rank 3.91), anxiety and rumination (75.5; mean rank 3.94), and irritability, conflict, and rage (74.7; mean rank 4.03) (Figure 2b). Suicidal thoughts and behaviors received the largest number of rank-1 selections (29/64), but attention/concentration and depressed mood/isolation showed the highest aggregate priority across the full rank distribution indicating broader consensus.

**Figure 3:**
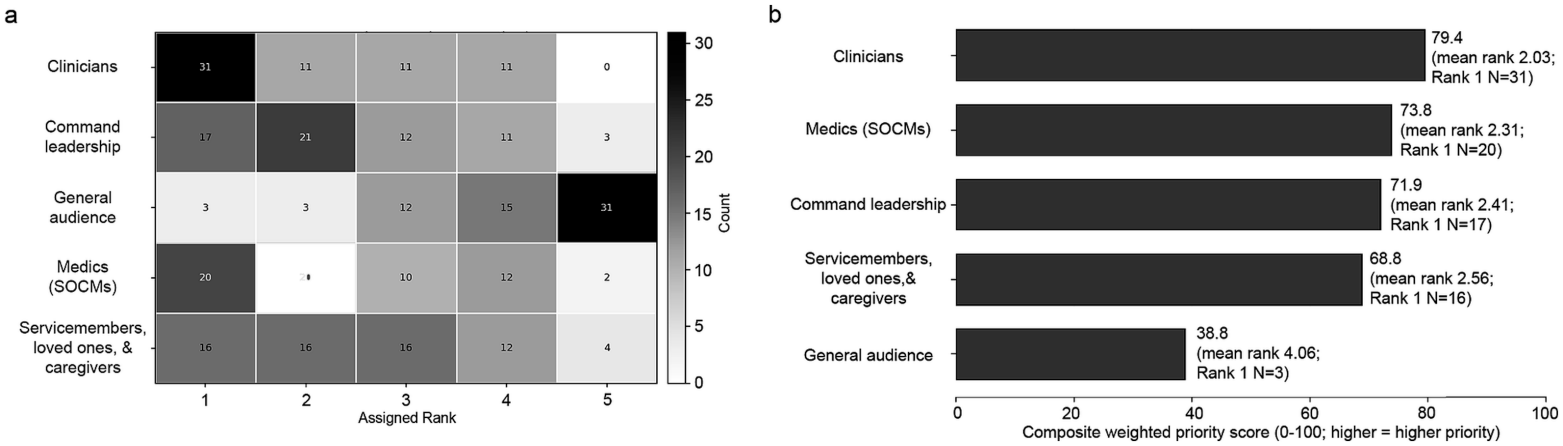
Stakeholder-endorsed audience priorities for research and informational products. **(a)** Publication-audience rank distribution heatmap (Rank 1 = highest priority). Grayscale annotated heatmap showing the rank counts for publication audience priorities. **(b)** Normalized Borda-style composite priority score, with higher values indicating higher priority; labels report mean rank and rank-1 counts. *N* = 64 respondents.

**Figure 4:**
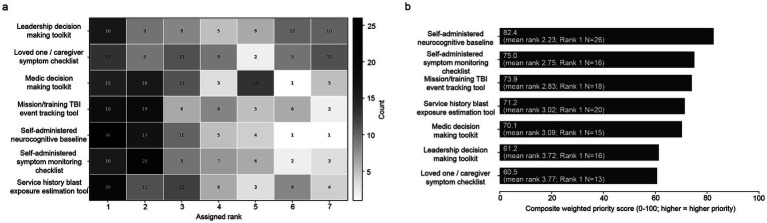
Stakeholder-endorsed tool priorities related to blast overpressure and TBI. **(a)** Tool rank distribution heatmap (Rank 1 = highest priority). Grayscale annotated heatmap showing the figure-derived rank counts used to summarize tool priorities. **(b)** Normalized Borda-style composite priority score, with higher values indicating higher priority; labels report mean rank and rank-1 counts. *N* = 64 respondents.

Summaries for publication audience (Figure 3) identified clinicians (79.4; mean rank 2.03; rank-1, *N* = 31) and medics (73.8; mean rank 2.31; rank-1, *N* = 20) as the highest-priority dissemination audiences, followed by command leadership (71.9; mean rank 2.41; rank-1, *N* = 17). Service members, loved ones, and caregivers occupied an intermediate position (68.8; mean rank 2.56; rank-1, *N* = 16), whereas the general audience was consistently deprioritized overall (38.8; mean rank 4.06; rank-1, *N* = 3) and was most often placed last (rank 5, *N* = 31). For tools priorities (Figure 4), self-administered neurocognitive baseline ranked highest (82.4; mean rank 2.23; rank-1, *N* = 26), followed by self-administered symptom monitoring checklist (75.0; mean rank 2.75; rank-1, *N* = 16), mission/training TBI event tracking tool (73.9; mean rank 2.83; rank-1, *N* = 18), service-history blast exposure estimation tool (71.2; mean rank 3.02; rank-1, *N* = 20), and medic decision-making toolkit (70.1; mean rank 3.09; rank-1, *N* = 15). The tool heatmap also suggests a distinction between high-consensus self-monitoring and exposure-documentation tools, which accumulated many top-1 to top-3 placements, and lower-priority support tools for leadership or loved ones/caregivers, which showed more diffuse rank distributions.

### Preferred educational formats

**Figure 5:**
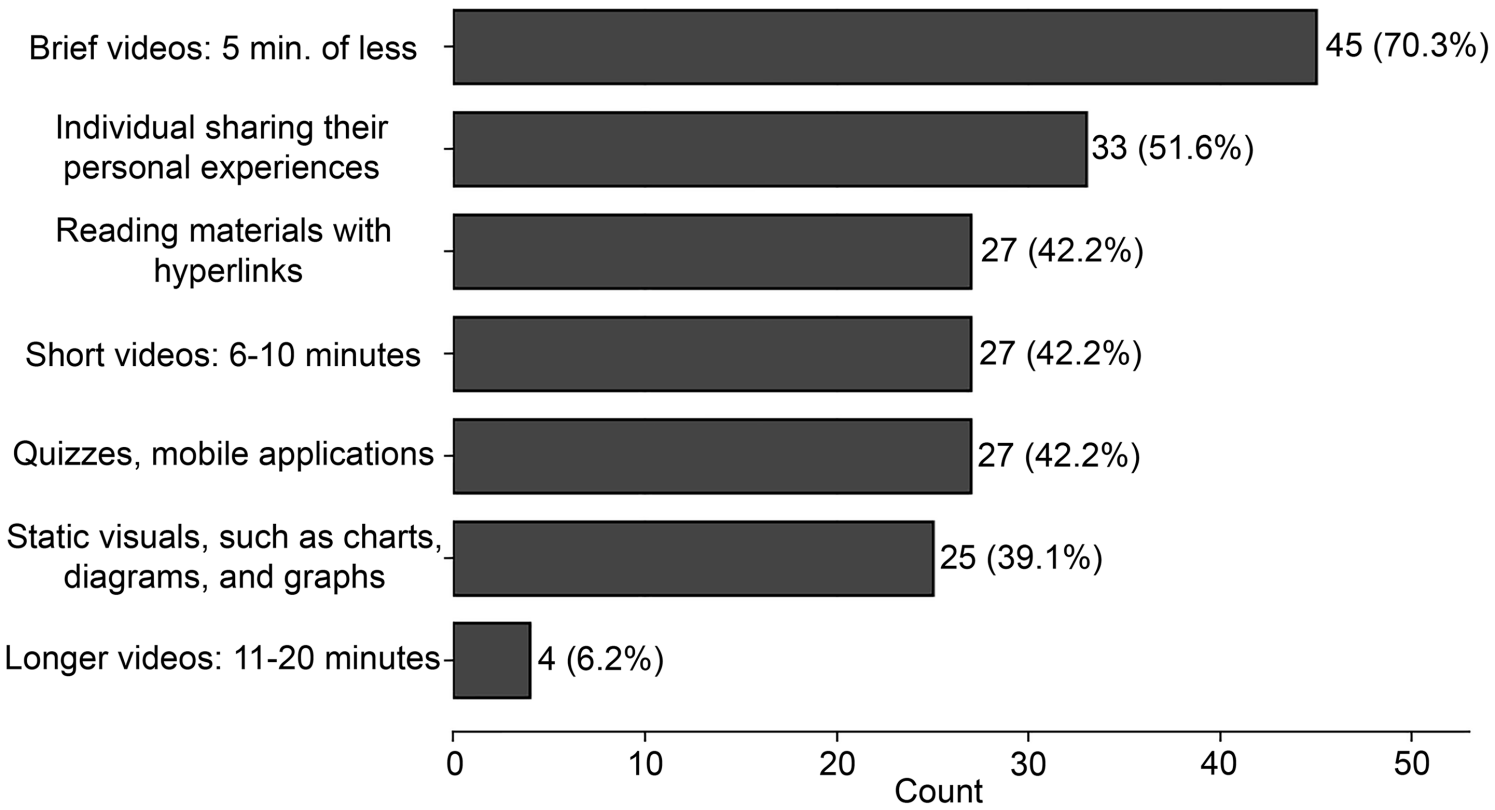
Stakeholder- preferred online information formats related to blast overpressure and TBI. Bar graph represent endorsement counts with percentage of respondents. *N* = 64; select all that apply.

When asked how they preferred to receive information through online educational tools, respondents most frequently selected brief videos of 5 min or less (45/64, 70.3%) and personal testimonials (33/64, 51.6%) (Figure 5). Short videos with subject-matter experts (6–10 min), quizzes or mobile applications, and reading materials with hyperlinks were each selected by 27/64 respondents (42.2%). Static visuals such as charts and diagrams were selected by 25/64 respondents (39.1%). Longer videos of 11–20 min were less commonly preferred (4/64, 6.3%).

## Discussion

This stakeholder survey of U.S. SOF community members identified pragmatic implementation gaps that appear actionable for near-term process improvement within Warfighter Brain Health efforts. Respondents emphasized provider knowledge and the credibility of first-touch care, access to specialized services, and reliable information that can support early recognition and symptom management. The prominence of medical knowledge gaps and limited access to specialized care aligns with DoD/W and VA priorities to standardize concussion and brain health management (1, 8, 9) and complements broader Warfighter Brain Health efforts to move from reactive, injury-triggered care toward routine brain health monitoring (1). Taken together, these findings argue SOF stakeholders are likely to have disproportionate gains from efforts involving education, information flow, and trusted pathways for early recognition and escalation at least during the early Warfighter Brain Health policy implementation phase.

Several findings may help guide implementation priorities. First, the prioritization of self-administered baseline and longitudinal symptom monitoring suggests that stakeholders value both an objective starting point and a simple mechanism for detecting change over time, particularly in cognition, depression, and suicidality. This emphasis is notable given established associations between mTBI and military suicide risk (15, 16). Second, the high priority assigned to TBI and blast exposure tracking tools underscores stakeholder demand for resources that can link training events to downstream symptoms and care decisions, consistent with emerging literature supporting cumulative blast exposure measures as predictors of symptom change (3, 6, 17). Third, the ranking of clinicians and medics as key dissemination targets indicates that educational resources should be tailored to the trusted gateway roles most likely to identify problems early and initiate escalation. Fourth, the frequency with which respondents highlighted family and intimate partner dynamics and the effects on family and loved ones supports family-forward dissemination (18) and caregiver tools (19) as readiness-relevant supports rather than ancillary additions.

The audience-ranking results further reinforce clinicians and medics as the most important early dissemination targets. Weighted results strengthened this pattern: clinicians and medics remained the top two priorities when the full rank distributions were aggregated, not only when rank-1 selections were counted. By contrast, the general audience was frequently ranked last, suggesting that respondents preferred dissemination strategies aimed first at groups with clearer responsibilities for recognition, triage, decision-making, and care navigation. In practical terms, stakeholders appear to want the people in first-touch and gateway care roles to be better prepared to recognize blast-related concerns, answer questions credibly, and facilitate timely referral or escalation. Because the survey did not ask respondents why they selected these audience categories, this interpretation remains provisional.

The tool and format findings also identify concrete areas for future development and testing. The high prioritization of self-administered neurocognitive baseline tools, self-administered symptom monitoring, and event- or exposure-tracking tools suggests demand for practical resources that help users detect change over time and connect symptoms to exposure history. Likewise, the preference for brief videos and testimonials suggests that short, accessible, experience-grounded educational formats may be more acceptable than longer didactic materials for at least some end users in this sample. These findings are best interpreted as candidate directions for co-design and pilot implementation rather than as definitive evidence that any single strategy will improve outcomes.

The weighted symptom summary suggests that respondents prioritized both cognitive and decision-making concerns and affective distress rather than a single narrowly defined symptom cluster. Attention, concentration, and decision-making changes and depressed mood and isolation had the highest aggregate priority scores across the full rank distributions, whereas suicidal thoughts and behaviors received the largest number of rank-1 selections. This pattern is consistent with a sample in which some respondents view suicidality as the single most urgent issue, while a broader portion consistently place cognitive and mood changes near the top of their priority lists. That distinction is important because it suggests dissemination materials may need to pair crisis-oriented content with earlier, more common indicators of functional decline that stakeholders perceive as actionable warning signs (15–17).

These findings are also consistent with broader DoD/W and VA efforts to strengthen standardized brain health management, improve blast-related education, and support earlier recognition of symptoms and care needs (1, 8–11). At the same time, the present data do not justify strong claims about population-wide preferences or policy mandates. A more appropriate next step is to use these findings to inform iterative resource development, stakeholder co-design, and prospective evaluation of whether specific educational products, tracking tools, or workflows are feasible, acceptable, and useful in operationally relevant settings.

This report has several limitations. First, the sample was small, convenience-based, and recruited through specific networks, which raises the possibility of sampling bias and limits external validity. Second, respondents were predominantly male and weighted toward active-duty members and Veterans with SOF backgrounds, while caregivers, spouses or partners, and other non-military stakeholders were comparatively underrepresented. Third, direct blast overpressure exposure history, mTBI history, and age were not collected or verified, which prevented exposure-stratified interpretation. Fourth, the survey instrument was purpose-built for this project and was not presented as a psychometrically validated measure. Fifth, weighted rank summaries were generated from aggregate rank data rather than from respondent-level data and therefore should be considered secondary descriptive estimates. Taken together, these factors mean the findings should be interpreted as a descriptive report of stakeholder input from a limited sample rather than as a representative characterization of all SOF community priorities.

Within these constraints, the survey provides useful directional guidance. The most consistent priorities support future work focused on improving clinician and medic knowledge, strengthening access to reliable and centralized information, developing pragmatic symptom-monitoring and exposure-documentation tools, and testing brief, role-appropriate educational formats. Those next steps should be pursued with broader recruitment, clearer characterization of respondent experience and exposure history, confirmation of the weighted rank results, and prospective evaluation of implementation impact.

### Public health implications

For high-tempo, high-exposure communities such as SOF, improved outcomes related to blast overpressure and mild traumatic brain injury may depend not only on clinical guidance but also on trusted workflows that fit operational constraints and reduce barriers to early recognition and care. In this stakeholder sample, the most consistent priorities supported investments in clinician- and medic-facing education, symptom management and monitoring tools, exposure documentation, and brief scalable educational formats that can reach service members, leaders, loved ones, and caregivers.

## Human participant protection

This report presents anonymized, aggregate survey findings to inform quality-improvement priorities related to blast exposure, mild traumatic brain injury, and Warfighter Brain Health efforts. The activity involved no prospective assignment to intervention and no evaluation of intervention effects on health-related biomedical or behavioral outcomes. Consistent with institutional determination, the work was classified as quality improvement / “not human subjects research” under the Emory University Quality Improvement “Special Operations Forces (SOF) Care Quality Improvement Survey” project (#4058). No directly identifying information was collected.

## Data Availability

The original contributions presented in the study are included in the article/Supplementary material, further inquiries can be directed to the corresponding author.
