# Supplementary material for: Stakeholder-informed priorities for process improvement in Warfighter Brain Health efforts
Source: Front Public Health. 2026 May 21;14:1815983. doi: 10.3389/fpubh.2026.1815983 (PMC13233381; doi:10.3389/fpubh.2026.1815983)
Supplement: Supplementary file 1 [file Supplementary_File_1.docx]

**Addendum**

The Bridging Research, Innovation, Diagnosis, Guidance, and Education for Special Operations (BRIDGES) survey used the following format:

---------BEGINNING OF SURVEY--------------

**1) Which of these best describes you (pick one):** Active-duty, Reservist or National Guard, Veteran (SOF background), Veteran (non-SOF background), Civilian

**2) Which of these best describe you? (select all that apply):** Current or former operator, Current or former enabler, Other current service member or veteran, Spouse or partner, Primary caregiver, Child of operator or enabler

**3) If applicable and if you’re willing to share, what was your MOS (military occupational specialty) or rating? We are asking this question to ensure that we receive responses from a variety of representatives within the SOF community:** [open input field]

**4) Which of these best describes your SOF service or affiliation:** US Air Force, US Army, US Coast Guard, US Marine Corps, US Navy, US Space Force

**5) In your opinion, what are the top FOUR (4) issues within your community related to blast exposure and mild TBI? Select 4:**

Balancing training requirements and health concerns

Getting blast/mild TBI effects information in a timely fashion

Community knowledge deficits

Immediate effects of blast and TBIs

Inability (or lack of access) to healthcare professionals who understand and know how to treat blast exposure effects

Lack of reliable and centralized information and education

Maintaining operational readiness while balancing adverse health effects

Medical knowledge deficits regarding blast exposure

Stigma around brain injuries

Effects on family and loved ones

**6) Based on your personal experience, please rank the order we should prioritize and publish educational information about blast/mild TBI, with #1 indicating we should publish education for that group first:** Clinicians: Physicians, nurses, dieticians, etc.; Command leadership; General audience; Medics (Special Operations Combat Medics; SOCMs); Service members, loved ones, & caregivers

**7) Based on your personal experience, please rank the blast/mild TBI symptoms we should address, with #1 indicating we should publish information on that symptom first:** Anxiety & rumination; Attention/concentration, time management, & decision making changes; Chronic brain changes; Depressed mood & isolation; Family & intimate partner dynamics; Sexual dysfunction & endocrine changes; Gastrointestinal problems; Slowed thoughts & movements; Headaches & chronic pain; Hearing, vision & balance changes; Irritability, conflict & rage; Suicidal thoughts & behaviors

**8) Based on your personal experience, please rank the blast/mild TBI tools we should publish in order, with #1 indicating we should publish that tool first:** Leadership decision-making toolkit; Loved one/caregiver symptom checklist; Medic/training TBI event tracking tool; Mission/training TBI event tracking tool; Self-administered neurocognitive baseline; Self-administered symptom monitoring checklist; Service history blast exposure estimation tool

**9) Based on your personal experience and the #1 choices you made for education, symptoms, and tools, please indicate which should we publish first:** Education; Symptoms; Tools

**10) When using online-based educational tools, how do you prefer to receive information? Select all that apply:**

Brief videos: 5 minutes or less

Short videos: 6 – 10 minutes with subject matter experts

Longer videos: 11 – 20 minutes with subject matter experts

Individual sharing their personal experiences

Reading materials with hyperlinks

Quizzes, mobile applications and static articles

Static visuals, such as charts, diagrams, and graphs

**11) In addition to publishing our care roadmap, which of the following would you like to see our team do in the coming 12 to 36 months? Select all that apply:**

Collaborate with branch-based leadership, including medical personnel

Establish a social media or website presence to share and disseminate information

Partner with social media-based Veteran Service Organizations to distribute results and information

Present the roadmap to US Army Special Operations Command leadership

Present the roadmap to individual organizations and Veteran Service Organizations

Present the roadmap to SOF medical personnel and professional organizations

Present the roadmap to spouse and family stakeholder groups

Present research and results to medical professional organizations and at medical conferences

VA needs educated

**12) Comments.** [open input field]

---------END OF SURVEY--------------
